# Pedunculoside alleviates cognitive deficits and neuronal cell apoptosis by activating the AMPK signaling cascade

**DOI:** 10.1186/s13020-024-01033-6

**Published:** 2024-11-22

**Authors:** Liwei Li, Jinfeng Sun, Fan Chen, Li Xiong, Lingyu She, Tang Hao, Yuqing Zeng, Luyao Li, Wei Wang, Xia Zhao, Guang Liang

**Affiliations:** 1 Zhejiang TCM Key Laboratory of Pharmacology and Translational Research of Natural Products, School of Pharmaceutical Sciences, Hangzhou Medical College, Hangzhou, 310014 Zhejiang China; 2 Department of Pharmacy and Institute of Inflammation, Zhejiang Provincial People’s Hospital, Affiliated People’s Hospital, Hangzhou Medical College, Hangzhou, 310014 Zhejiang China; 3 Key Laboratory of Natural Medicines of the Changbai Mountain, Ministry of Education, Yanbian University, Yanji, Jilin 133002 People’s Republic of China; 4 Affiliated Yongkang First People’s Hospital, Hangzhou Medical College, Yongkang, 321399 Zhejiang China; 5 School of Pharmaceutical Sciences, Wenzhou Medical University, 1210 University Town, Wenzhou, 325035 Zhejiang China; 6 School of Pharmacy, Hangzhou Medical College, Hangzhou, 311399 Zhejiang China

**Keywords:** Alzheimer’s disease, Pedunculoside, 3 × Tg-AD mice, Oxidative stress, Apoptosis

## Abstract

**Background:**

Mitochondrial dysfunction emerges as an early pathological hallmark of Alzheimer's disease (AD). The reduction in mitochondrial membrane potential and the elevation of reactive oxygen species (ROS) production are pivotal in the initiation of neuronal cell apoptosis. Pedunculoside(Ped), a novel triterpene saponin derived from the dried barks of *Ilex rotunda Thunb*, exhibits a potent anti-inflammatory effect. In the course of drug screening, we discovered that Ped offers significant protection against apoptosis induced by Aβ1-42. Nevertheless, the role and mechanism of Ped in AD are yet to be elucidated.

**Methods:**

Oxidative stress was evaluated by measuring mitochondrial membrane potential and intracellular ROS production. The expression of proteins associated with apoptosis was determined using western blot analysis and flow cytometry. In vivo*,* the pathological characteristics of AD were investigated through Western blot and tissue immunofluorescence techniques. Cognitive function was assessed using the Morris Water Maze and Novel Object Recognition tests.

**Results:**

We demonstrated that Ped decreased apoptosis in PC12 cells, reduced the generation of intracellular ROS, and restored mitochondrial membrane potential. Mechanistically, we found that the protective effect of Ped against Aβ-induced neurotoxicity was associated with activation of the AMPK/GSK-3β/Nrf2 signaling pathway. In vivo*,* Ped alleviated memory deficits and inhibited neuronal apoptosis, inflammation, and oxidative stress in the hippocampus of 3 × Tg AD mice, along with the activation of the AMPK signaling pathway.

**Conclusion:**

The findings indicate that Ped exerts its neuroprotective effects against oxidative stress and apoptosis through the AMPK signaling cascade. The results demonstrate that Ped is a potential candidate for the treatment of AD.

**Graphical Abstract:**

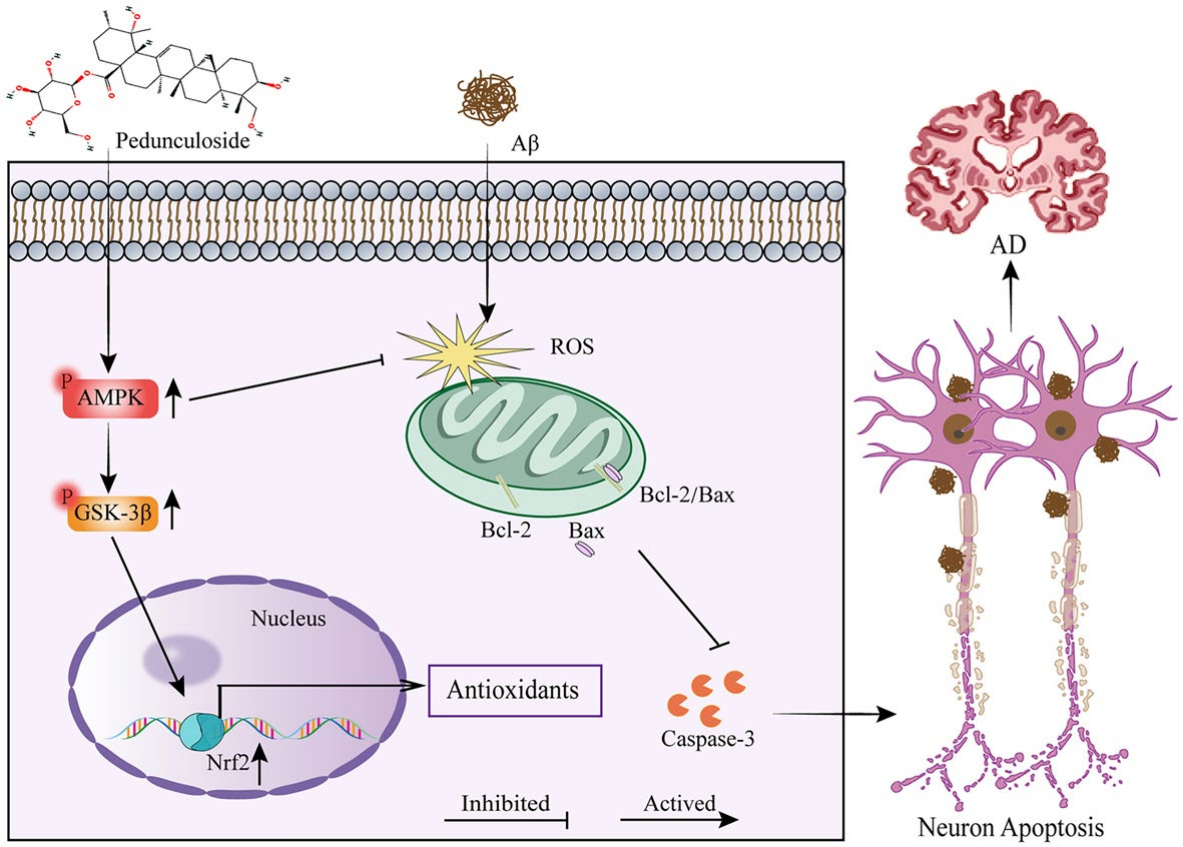

**Supplementary Information:**

The online version contains supplementary material available at 10.1186/s13020-024-01033-6.

## Introduction

Alzheimer's disease (AD) is the most common form of dementia, accounting for 60%-70% of all patients [1]. The two main pathological characteristics of AD are the deposition of beta-amyloid (Aβ) plaques and hyperphosphorylated tau protein-caused neurofibrillary tangles (NFTs) [2]. Based on the pathological features exhibited by AD, researchers have proposed several hypotheses, including the cholinergic hypothesis, the Aβ cascade hypothesis, the tau protein abnormal phosphorylation hypothesis, the neuroinflammatory hypothesis, and the metal ion dysregulation hypothesis [1]. The Aβ cascade hypothesis has been widely recognized, according to which abnormal extracellular accumulation of Aβ triggers the formation of senile plaques in the hippocampal brain region of the brain, which subsequently triggers a series of pathological processes, including oxidative stress and apoptosis [3]. Report suggests that Aβ-induced oxidative stress may be critical for AD progression [4]. Oxidative stress refers to a condition characterized by an imbalance between the generation and removal of reactive oxygen species (ROS), leading to an impaired equilibrium between oxidation and antioxidation. Mitochondrial dysfunction, as a primary contributor to the production of ROS, and a key target of oxidative damage, has been associated with both the aging process and the development of AD [5]. Therefore, developing multi-target drugs against oxidative stress may be an effective strategy [6–8].

AMP-activated protein kinase (AMPK) plays an important role in regulating energy metabolism. It is the main pathway that regulates mitochondrial function as well as oxidative stress and regulatory role in apoptosis caused by mitochondrial dysfunction [9]. Nuclear factor erythroid 2-related factor 2 (Nrf2), is a key transcription factor that regulates antioxidant stress, plays an important role in the body's antioxidant response, such as regulating redox balance, DNA repair, and mitochondria function [10]. AMPK can regulate GSK-3β and affect Nrf2 nuclear protein levels. Studies showed that the AMPK pathway is involved in the pathogenesis of AD. The AMPK/GSK-3β/Nrf2 system has emerged as an important therapeutic target for the treatment of AD [11]. Activating AMPK can inhibit oxidative stress, improve mitochondrial function and sugar uptake, reduce Aβ aggregation, and help slow down the formation of Aβ plaques [12, 13]. Therefore, targeting the AMPK/GSK-3β/ Nrf2 pathway may have potential treatment in AD.

Traditional Chinese Medicine has garnered growing attention in recent years. It is considered a viable treatment option for a variety of diseases [11], including diabetes, obesity, cancer, infectious diseases, metabolic syndrome, and neurological disorders [14]. We conducted a screening of the Natural Product Library obtained in the laboratory, initially obtained Pedunculoside (Ped), a natural product with neuroprotective effects. Ped is the main biologically active ingredient in the traditional Chinese herbal medicine "*Ilicis Rotundae Cortex*", a triterpenoid [15]. Modern pharmacology shows that Ped has a variety of pharmacological activities [15–19]. Triterpenoids have been studied for their therapeutic effects in AD [20] and Ped has an anti-inflammatory effect [17], whereas the same chronic inflammatory response exists in AD. So we hypothesized that Ped may have a therapeutic role in AD and this has not yet been reported.

In this study, we investigated the preventive and therapeutic efficacy of Ped in in vitro and in vivo AD models and elucidated its potential mechanism of action. The results indicate that Ped is a potential therapeutic drug for the prevention and treatment of AD.

## Material and methods

### Reagents

Aβ (1–42) was purchased from Ontores Biotechnologies (PA4391). Peduncloside (42719-32-4, 98% purity) was obtained from Chengdu DeSiTe Biological Technology (DJ0034). Selectable Natural Product Library was obtained from TargetMol library service. Donepezil (110119-84-1, 98% purity) was obtained from Chengdu Alfa Biotechnology. Dimethyl sulfoxide (DMSO; D2650) and Dulbecco’s modified Eagle’s medium (DMEM; D1152) were ordered from Sigma (St. Louis, MO, USA). 0.25% Trypsin (BC-CE-005), DAPI buffer (4, 6-diamidino-2-phenylindole; C0065), bovine serum albumin (BSA; A8020), MTT powder (M2128), JC-1 assay kit (C2005), RIPA sample lysis buffer (P0013B) and ROS assay kit (S0033) were purchased from Beyotime Biotechnology Institute. 100 × Penicillin–Streptomycin Solution (Double antibody; 15140–122). Annexin V-FITC/PI Apoptosis Detection Kit was purchased from BD Biosciences (556547). Triton X-100 (P1080) were ordered from Solarbio. Compound C (HY13418A) was bought from MCE (MedChemExpress). PVDF membrane (SEQ00010) were obtained from BIO-RAD. A list of the antibodies used in the experiments can be found in Table S1. Natural product library information was listed in Table S2.

### Cell culture

PC12 cells are a cell line derived from rat pheochromocytoma and are commonly used in neurobiological studies due to their neuron-like characteristics. PC12 cells were cultured in DMEM medium with 10% FBS, and 1 × Penicillin–Streptomycin antibody added. Cells were incubated at 37 °C in a 5% CO_2_ cell culture incubator. The cell culture medium is changed every 1 to 2 days and cell passage is performed every 2 to 3 days.

### Aβ oligomer preparation

Aβ plaques are the major extracellular constituent in AD, produce neurotoxicity, and induce neuronal damage ultimately leading to neuronal death [21–23]. In this study, an Aβ stock solution was prepared with sterile dimethyl sulfoxide at a concentration of 10 mM, and aliquots were properly stored at −20 °C. As previously indicated, Aβ was subjected to incubation at 37 °C for 7 days to promote aggregation before experimental use [24, 25]. During the experimental procedures, Aβ was diluted to the desired concentration with a culture solution.

### MTT assay

Preparation of MTT: 0.5 g of MTT dissolved in 100 mL of PBS at a storage concentration of 5 mg/mL. Ensure complete dissolution and pass the solution through a 0.22 μM filter membrane to eliminate bacteria. Store the filtered solution at −20 ℃ in a light-free environment. Briefly, the cells were cultured in a complete medium with a seeding density of 7 × 10^3^ cells per well in a 96-well plate. Following respective treatments, the cells were incubated with MTT (0.5 mg/mL) for an additional 2 to 3 h. Subsequently, the blue formazan crystals that were produced by live cells were dissolved with 100 μL DMSO, and the resulting absorbance was measured at 490 nm using a Spectra Max 250 microplate reader (Molecular Device, Sunnyvale, CA, USA). Finally, cell viability was calculated using the control group as a reference.

### Measurement of reactive oxygen species (ROS)

Intracellular ROS were detected using a ROS kit according to the protocol provided by Beyotime. After drugs treatment, DMEM medium containing ROS was added to each group and incubated at 37 °C for 1 h. Then, wash 3 times with PBS. Qualification of fluorescence intensity was measured using an Infinite M200 PRO multi-mode microplate reader (excitation: 488 nm and emission: 525 nm).

### Detection of mitochondrial membrane potential (△ψm)

ΔΨm was assayed using the JC-1 Mitochondrial Membrane Potential Assay Kit according to the instructions provided by the manufacturer. After drugs treatment, DMEM medium containing JC-1 was added to each group. Incubate at 37 °C for 1 h, and then wash three times with PBS solution. The red fluorescence (emission at 595 nm, excitation at 560 nm) and green fluorescence (emission at 535 nm, excitation at 485 nm) were quantified using Multimode Microplate to determine their respective intensities. The ΔΨm was then calculated by analyzing the ratio of red to green fluorescence intensities.

### Flow cytometry

PC12 cells were seeded into 12-well plates at a density of approximately 5 × 10^5^ cells/well. After drug treatment, all cells were collected and centrifuged at 1000 rpm for 5 min. After washing with PBS, the cells were suspended in 195 μL of Annexin V-FITC/PI binding buffer. Subsequently, 5 μL of Annexin V-FITC was added and incubated for 15 min at room temperature in the dark, followed by 10 μL of propidium iodide (PI) and incubated for 5 min at room temperature in the dark. Cells were then collected and analyzed using BD Accuri^™^ C6 Plus.

### Animal and drug administration

3 × Tg-AD mice (PS1_M146V_, APP_Swe_, and Tau_P301L_ transgenic) with similar weight and age were procured from the animal facility of Hangzhou Medical College and bred in Hangzhou Medical College animal facility. The mice were accommodated in laboratory conditions that met the standard requirements for specific pathogen-free facilities. Provide them with adequate food and water, and each cage housed 8 mice. Eight-month-old female 3 × Tg-AD mice were randomly divided into five groups: Wild-type (WT); 3 × Tg-AD; 3 × Tg-AD mice treated with 5 mg/kg Ped (3 × Tg-AD + 5 mg/kg Ped); 3 × Tg-AD mice treated with 10 mg/kg Ped (3 × Tg-AD + 10 mg/kg Ped), and 3 × Tg-AD mice treated with 5 mg/kg donepezil (3 × Tg-AD + 5 mg/kg Don). All drugs in the experiment were dissolved in PBS containing 2% dimethyl sulfoxide (DMSO), and the control group was injected with an equal volume of PBS containing 2% DMSO. Before drug administration, the mice were weighed and injected intraperitoneally based on their respective body weights. The administration of intraperitoneal injections was performed once a day for one month across all experimental groups.

### Test of novel object recognition (NOR)

To assess the mice's memory and learning ability, we performed the NOR test. On the first day, two identical circular objects A were placed in the test arena (50 cm L × 50 cm W × 40 cm H). Each mouse was given 5 min to freely explore the objects. Then after 24 h, one of the round objects A was randomly replaced with a square object B and each mouse was given 5 min to explore freely. The exploratory behavior was counted as the mice pointing their noses to objects within 2 cm or touching the objects directly with their noses and/or front paws. The discrimination index was calculated from the time spent exploring the new object and the total number of explorations.

### Test of Morris water maze (MWM)

The learning and memory abilities of all mice were examined using the MWM test. In the MWM experiment, groups of mice were first trained 3 times to let them know the location of the platform in the tank. The mice were then subjected to 4 days of navigation tests, during which they were required to find a hidden platform located 1 cm underwater. Each mouse was subjected to 2 trials, each of which lasted 60 s with a 60 s interval between each trial. Experimental data were recorded using Visu Track from Shanghai Xin Ruan Soft Information Technology Co. On the fifth day, the platform was removed from the water and the mice were allowed to swim freely for 60 s to conduct a space exploration experiment.

### Tissue samples preparation

After completion of the behavioral assessments, all mice were humanely euthanized via administration of Pentobarbital sodium (50 mg/kg). Before removing the brain tissues, the mice were perfused with cold PBS, then slowly injected with 20 mL of 4% paraformaldehyde solution (0.01 mol/L PBS, pH 7.40, 4 °C), and finally, the mice were neck-broken and executed. Removed portions of the samples were dehydrated through a sucrose gradient and embedded using O.C.T. and then stored at −80 °C for subsequent analysis.

### Immunofluorescent staining (IF)

O.C.T.-embedded brain tissue was sectioned into 20 μm using a cryosectioner (Leica, CM1950). After permeabilization and sealing steps, the tissue was incubated overnight at 4 °C using the corresponding primary antibody. The next day, incubate with Alexa Fluor-conjugated secondary antibody for 1 h at room temperature. Subsequently, the sections were mounted using slow fade^®^ anti-fade DAPI reagent and images were captured using a Nikon A1 confocal microscope. All experimental procedures were repeated three times to ensure consistency and statistical robustness.

### Western blot assays

Proteins are extracted from cell or tissue homogenates using RIPA (strong) buffer containing a mixture of protease and phosphatase inhibitors. The protein concentration of the extracted samples was determined using the BCA Protein Quantification Kit and boiled in a metal bath for 10 min after adding the appropriate amount of upwelling buffer. To facilitate the separation of proteins, SDS-PAGE was prepared with the addition of 40 μg of protein. The proteins were then separated using electrophoresis, employing a sampling gun. Subsequently, the separated proteins were transferred onto a PVDF membrane using a wet transfer method. After the completion of electrotransfer, the PVDF membrane strips were blocked with a 3% BCA solution for 1 h. The strips were incubated overnight at 4 °C using the primary antibodies in Supplementary Table S1. The immunoreactive bands were detected using the Enhanced Chemiluminescence Kit, and the gray values of the bands were quantified using Image J software.

### Statistical analysis

Statistical analysis was conducted utilizing the GraphPad Prism 8 software. The mean ± SEM of results were derived from at least three independent tests. The statistical significance among multiple groups was evaluated using one or two-way ANOVA, subsequently followed by Tukey’s post-hoc test. The threshold of p < 0.05 was considered statistically significant.

## Results

### Ped decreased Aβ-induced apoptosis in PC12 Cells

**Fig. 1:**
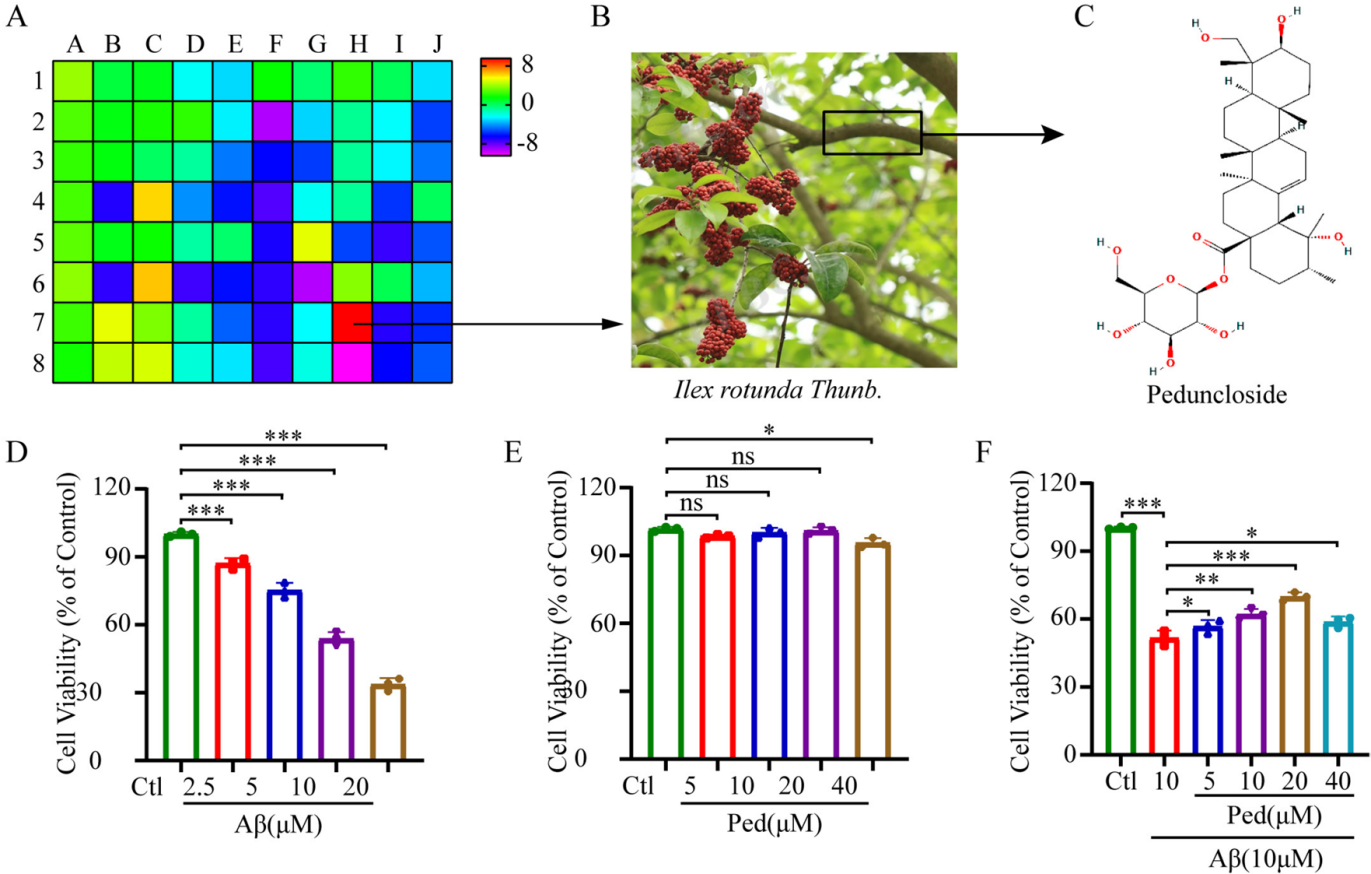
Ped alleviates Aβ-induced reduction in PC12 cell viability. We screened 80 compounds from natural chemical libraries to explore their protective effects against Aβ-induced injury in PC12 cells. **A** The heatmap shows the amount of cell viability restoration using natural products. Ped was identified as the best candidate drug. **B** Plant: *Ilex rotunda Thunb*. Image from Plant Photo Bank of China (PPBC). **C** Chemical structure of Ped. **D** Aβ concentration screening. After treatment of PC12 cells with different concentrations of Aβ, cell viability was assayed using the MTT assay to determine the concentration of Aβ used. **E** Ped concentration gradient screening. **F** Pharmaco-protective concentration screening. PC12 cells were pretreated with different doses of Ped for 2 h and then exposed to Aβ for another 24 h. Cell viability was determined using MTT assay. *ns*—not significant, *p < 0.05, **p < 0.01, ***p < 0.001. n = 3 independent experiments

**Fig. 2:**
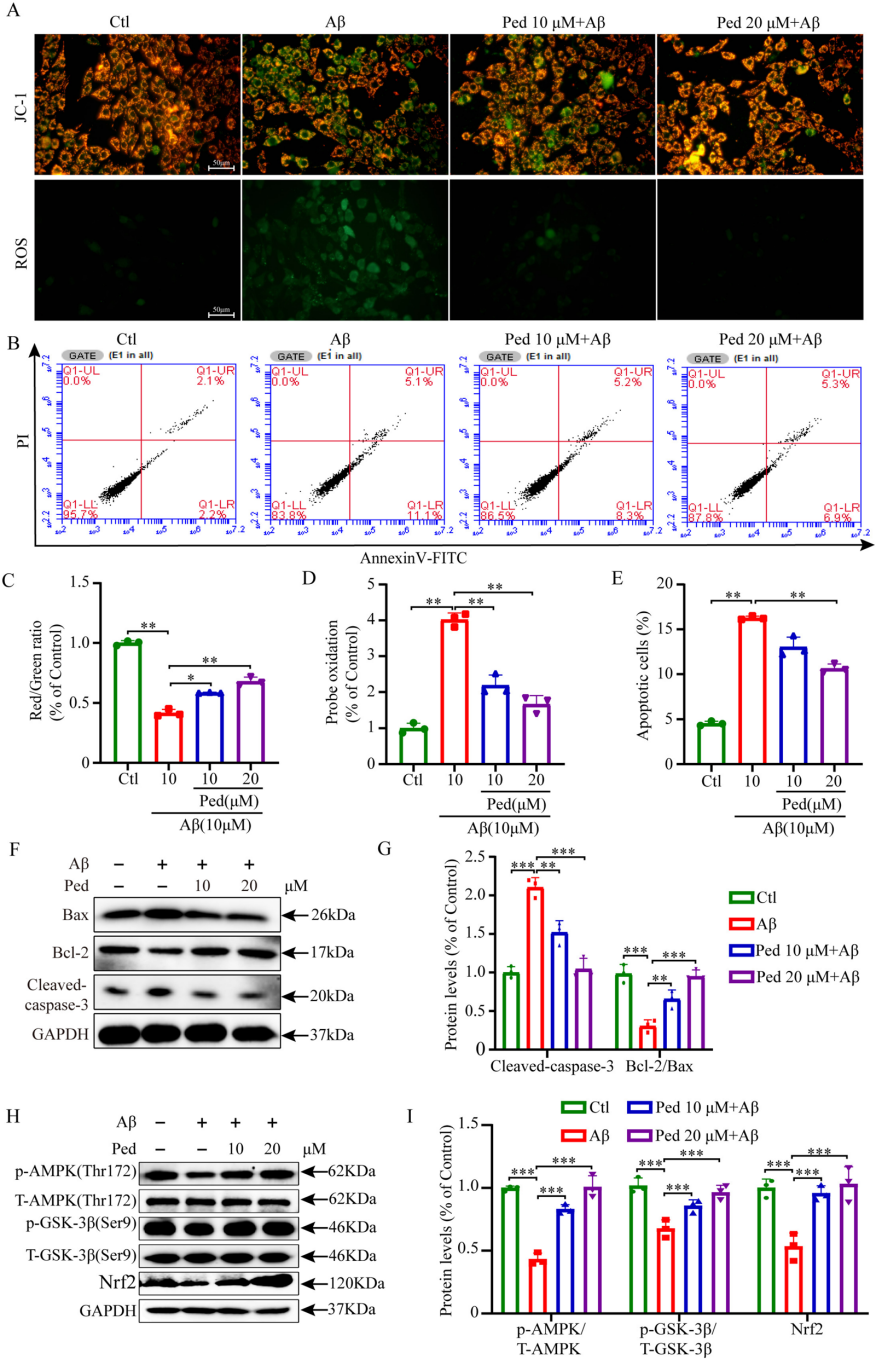
Ped decreased Aβ-induced apoptosis in PC12 cells. **A** Oxidative stress were evaluated by JC-1 and ROS staining in PC12 cells. **B** Apoptosis was detected using flow cytometry. **C** Quantitative analysis of JC-1 staining in A. Aβ treatment significantly reduced the ratio of red fluorescence to green fluorescence, while Ped treatment significantly increased the ratio of red fluorescence to green fluorescence. **D** Quantitative analysis of ROS staining in A. Aβ treatment significantly increased green fluorescence, while administration of Ped protection significantly decreased green fluorescence. **E** Quantitative analysis of flow cytometry result in B. Ped pre-protection significantly reduced Aβ-induced apoptosis in PC12 cells. **F** Western blot analysis of Cleaved- caspase-3, Bax, and Bcl-2 in PC12 cells. GAPDH was used as the loading control. **G** Quantitative data of Cleaved- caspase-3, Bax, Bcl-2 blot, and Bcl-2/Bax ratio intensity determined by ImageJ software. **H** Expression of p-AMPK, T-AMPK; p-GSK-3β, T-GSK-3β, and Nrf2 were detected by using Western blot. **I** Quantification of p-AMPK/T-AMPK, p-GSK-3β/T-GSK-3β and Nrf2 in H. *ns*—not significant, *P < 0.05 or **P < 0.01, or ***P < 0.001. n = 3 independent experiments

To explore natural compounds with neuroprotective effects, we screened a library of 80 natural compounds purchased from TargetMol library service for their effects on Aβ-induced neurotoxicity by MTT assay. The obtained results indicate that Ped has the best protective effect among those compounds against Aβ-induced PC12 cell damage (Fig. 1A). Therefore, we chose to take Ped as the object of an in-depth study. Ped is the key active ingredient in *Ilicis Rotundae Cortex*, which is extracted from the bark of the *Ilex rotunda Thunb* (Fig. 1B). The structural formula for Ped is shown in Fig. 1C. To further investigate the protective effect of Ped, we pre-protected PC12 cells using 4 concentrations of Ped, which were also assayed using the MTT method. The results showed that the cell viability in the model group was significantly decreased, while the cell viability was significantly improved by using 10 and 20 μM drug concentrations (Fig. 1F,). Similarly, AnnexinV-FITC/PI -positive cells were further analyzed through flow cytometry. The experimental findings revealed a significant increase in PC12 cell apoptosis following Aβ exposure. However, pretreatment with 20 μM Ped demonstrated a significant reduction in Aβ-induced apoptosis (Fig. 2B).

Oxidative stress was widely recognized as a pivotal factor in initiating and worsening the pathogenesis of Aβ induced cellular damage [26]. To assess the impact of Ped on oxidative stress we performed ROS staining. As expected shown in Fig. 2A, following the induction of Aβ, there was an increase in intracellular ROS production. Pretreatment with Ped attenuated the elevated ROS levels induced by Aβ. Consequently, we can preliminarily infer that Ped has the capacity to attenuate the generation of intracellular ROS, suggesting its potential as an inhibitor of free radicals. To assess the integrity of mitochondrial function, the mitochondrial membrane potential (ΔΨm) was assessed by employing the JC-1 probe. Based on the findings elucidated in Fig. 2A, it is evident that the exposure of PC12 cells to Aβ stimulation leads to a considerable decline in mitochondrial membrane potential (indicated by the red-green fluorescence ratio). Conversely, the application of the Ped exhibits a concentration-dependent effect by bolstering the mitochondrial membrane potential.

Western blot analysis of apoptotic protein expression demonstrated that exposure to Aβ resulted in a marked elevation in the levels of the pro-apoptotic protein Bax, accompanied by a substantial suppression of the anti-apoptotic protein Bcl-2. Notably, these effects were reversed by pre-treatment with Ped (Fig. 2F). Caspase-3 is considered a key enzyme leading to apoptosis, and its activation heralds the beginning of the execution phase of apoptosis. Therefore, we examined the expression of Cleaved-caspase-3 using Western blot and result showed that Ped significantly inhibited the expression of Cleaved-caspase3 protein in PC12 cells induced by Aβ induction.

### Involvement of AMPK in the neuroprotection of Ped

AMPK, a ternary Ser/Thr protein kinase composed of α, β, and γ subunits, is a major regulator of cellular energy homeostasis [27], as well as an important sensor for monitoring cellular energy status, central to glucose and lipid metabolism, and implicated in the pathogenesis of AD [28]. There is growing evidence that AMPK has a regulatory role in all aspects of mitochondrial biology and homeostasis. Numerous studies have shown that increasing neuronal energy may provide beneficial effects in neurodegenerative diseases. AMPK is a key sensor of cellular energy status and an upstream regulator of multiple cellular mechanisms, it plays a crucial role in AD processes [29]. Nrf2 is downstream of AMPK and is also a regulator of mitochondrial membrane potential regulation and oxidative stress homeostasis [30]. GSK-3β is also located downstream of AMPK and it has been shown that overactivation of GSK-3β leads to abnormal energy metabolism and ultimately neuronal death, especially in AD [31]. To further explore the involvement of the AMPK cascade in the protective effects of Ped, we examined the AMPK/GSK-3β/Nrf2 signaling pathway. We conducted Western blot analyses to assess the expression levels of p-AMPK, p-GSK-3β, and Nrf2.

The experimental findings suggest that treatment of PC12 cells with Aβ significantly decreased the expression levels of p-AMPK, p-GSK-3β, and Nrf2 proteins (Fig. 2H). Phosphorylation of AMPK occurs mainly at Thr172 of the α-subunit, so we phosphorylated AMPKα (Thr172). Consistent with our expectations, treatment with Aβ led to a significant reduction in AMPK phosphorylation compared to the control group. However, pre-treatment with Ped resulted in a remarkable increase in AMPK phosphorylation, even surpassing the level observed in the control group (Fig. 2H). Next, we aim to investigate the correlation between the phosphorylation activity of GSK-3β(ser9) and the neuroprotective impact of Ped. As anticipated and in agreement with previous findings, the phosphorylation of GSK-3β(ser9) exhibited a notable reduction in PC12 cells treated with Aβ when compared to the control group. In sharp contrast, a striking rise in GSK-3β phosphorylation was observed following pre-treatment with Ped, reaching levels comparable to the phosphorylation observed in untreated control cultures (Fig. 2I). Similarly, the level of Nrf2 protein expression was significantly increased after drug administration (Fig. 2I). This indicates that Ped may function via the AMPK/GSK-3β/Nrf2 signaling pathway.

**Fig. 3:**
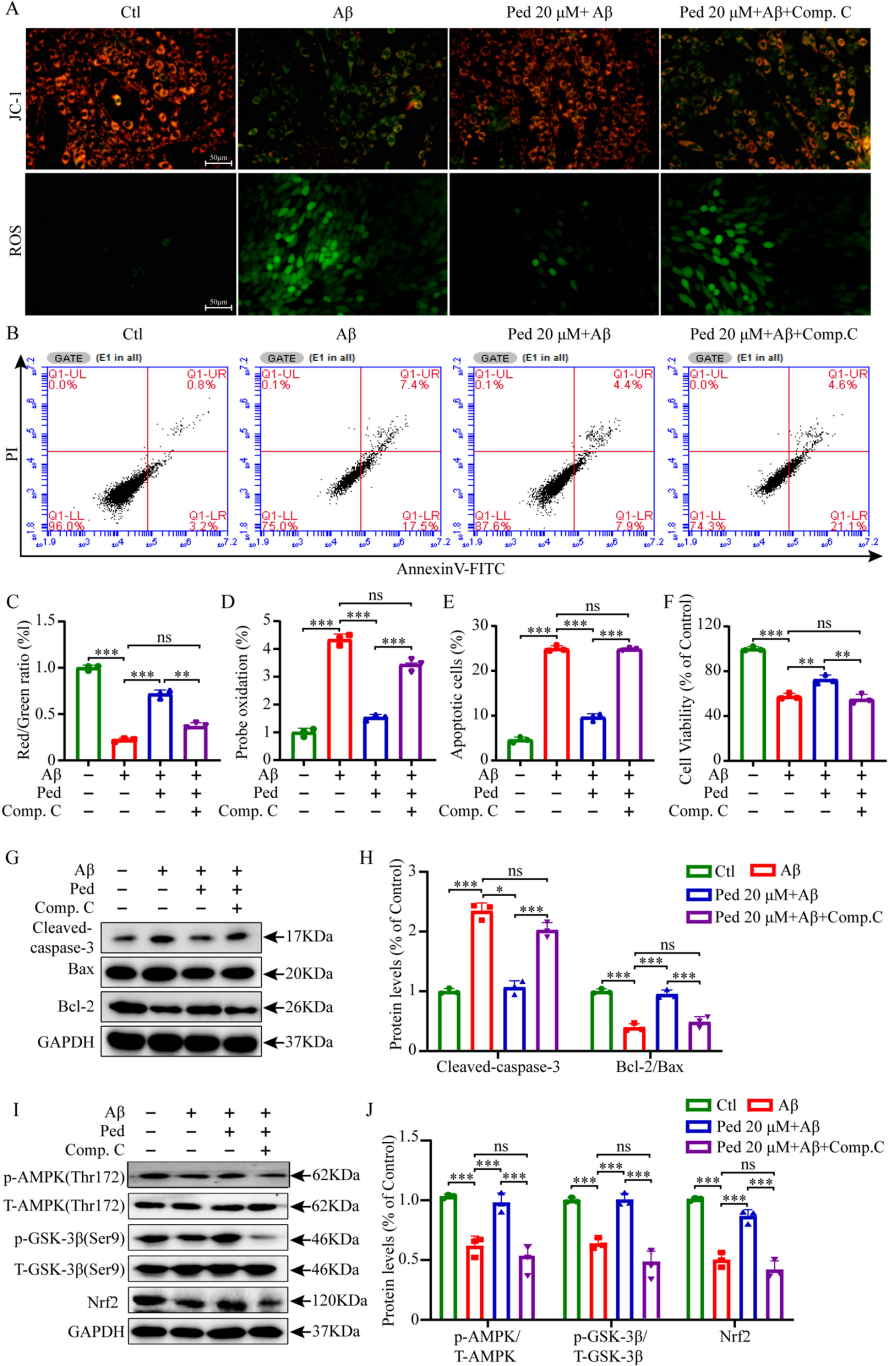
Ped reduces Aβ-induced apoptosis in PC12 cells through the AMPK signaling cascade. PC12 cells in the inhibitor group were pretreated with 5 μM Compound C (AMPK inhibitor) for 30 min, and then each group of cells were treated with 20 μM Ped for 2 h, and then incubated with 10 μM Aβ (Aβ group, Ped group, inhibitor group) for another 24 h. **A** Images of JC-1 and ROS staining in PC12 cells. **B** Apoptosis was detected using flow cytometry. **C**-**D** Quantitative analysis of JC-1 and ROS staining in A. **E** Quantitative analysis of flow cytometry in B. **F** Cell viability was measured by MTT assay. **G** Western blot analysis of Cleaved-caspase-3, Bax, and Bcl-2 in PC12cells. GAPDH was used as the loading control. **H** Quantitative analysis of protein levels in G. **I** Western blot analysis of p-AMPK, T-AMPK; p-GSK-3β, T-GSK-3β, and Nrf2 in PC12 cells. **J** Quantification of protein levels in K. *ns*—not significant, *P < 0.05 or **P < 0.01, or ***P < 0.001. n = 3 independent experiments

Compound C, also known as dorsomorphin, is an exceptionally potent, cell-permeable compound that selectively blocks AMPK activity by competitively binding to ATP. Therefore, we subsequently used Compound C to evaluate whether Ped exerts neuroprotective effects via modulation of the AMPK signaling pathway. In subsequent experiments, we chose to pre-treat the cells with Compound C, then give Ped, and further incubate with Aβ. Next, cell apoptosis was assessed via flow cytometry (Fig. 3B), and mitochondrial damage was evaluated using JC-1 and ROS staining (Fig. 3A). Cell viability was quantified through MTT assays (Fig. 3F), In addition, we determined the expression of apoptosis protein-related proteins Bax, Bcl-2, and Cleaved-caspase-3(Fig. 3G), as well as the phosphorylation activity of the pathway-related protein AMPK/GSK-3β (ser9), and the expression of Nrf2 using the Western blot method (Fig. 3I). JC-1 and ROS staining according to Fig. 3C and D showed that pretreatment with Compound C reversed the protective effect of Ped against oxidative stress. Flow data in Fig. 3B showed a significant increase in apoptotic cells after blocking the pathway using Compound C. Figure 3G and I Western blot results showed that Compound C partially reversed the inhibitory effect of Ped on apoptosis and its activation of the AMPK/GSK-3β/Nrf2 signaling pathway. The results above unequivocally demonstrated that pretreatment with Compound C significantly reduced the neuroprotective effects of Ped (Fig. 3A-J).

### Ped attenuated spatial learning and reference memory deficits in 3 × Tg-AD mice model

**Fig. 4:**
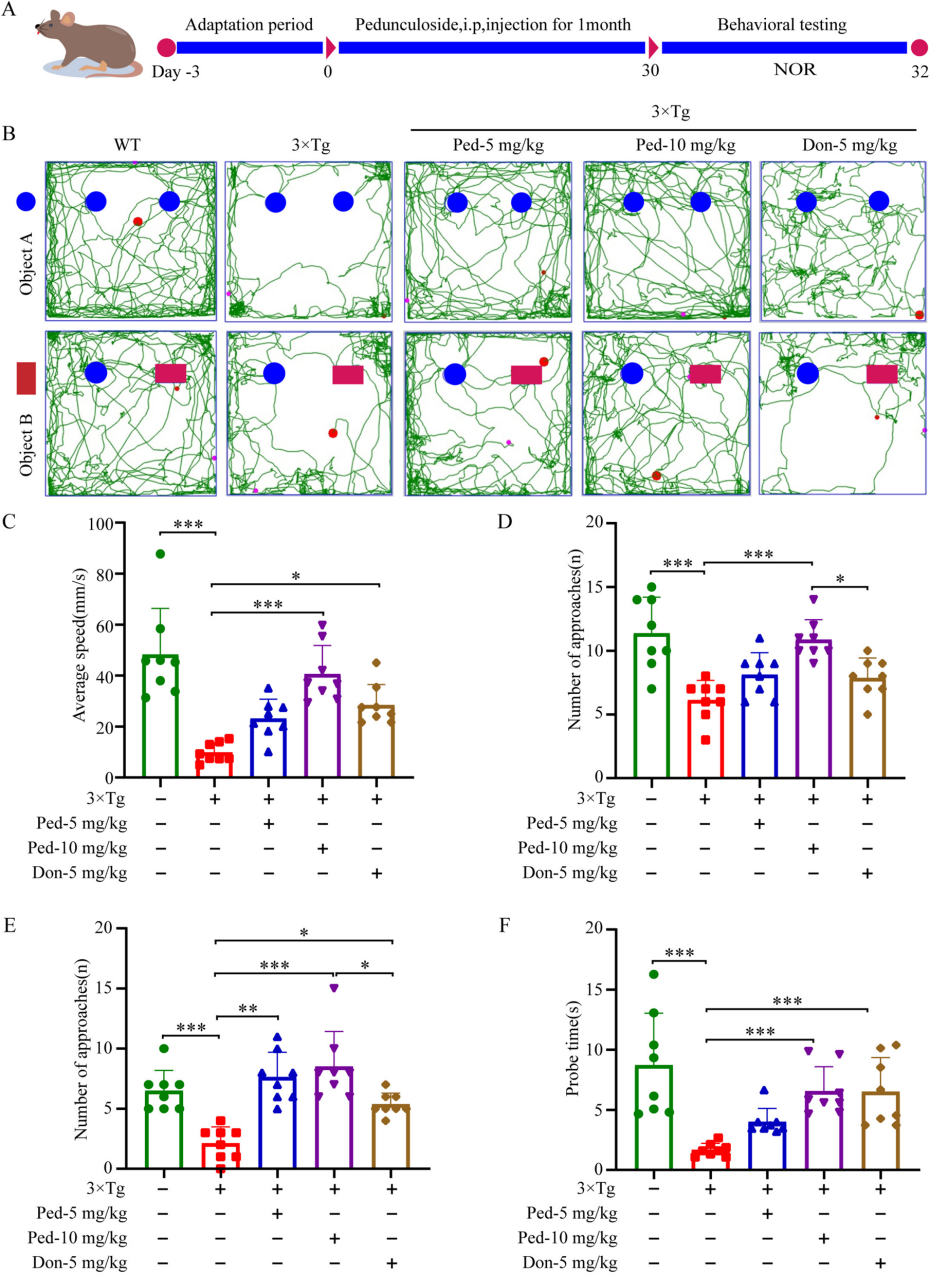
Ped alleviates the cognitive impairment in 3 × Tg-AD mice. **A** Ped improved learning and memory in 3 × Tg-AD mice. 8–month-old 3 × Tg-AD mice were administered with Ped or by intraperitoneal injection once a day for one month. **B** NOR experiment trajectory diagram. **C** On the first day, the average speed of the mice's movements. **D** The number of times the mice explored the object on the first day. **E** The next day, the mice explored the new object a number of times. **F** The next day, the mice spent time exploring new objects. *ns*—not significant, *P < 0.05 or **P < 0.01, or ***P < 0.001. n = 10 mice/Group

Based on the results of the above in vitro studies, we next verified whether the neuroprotective effects of Ped in PC12 cells could play a role in a triple transgenic mice model of AD (3 × Tg-AD). 3 × Tg-AD transgenic mice are a type of mice whose age leads to increasing accumulation of Aβ and changes in extracellular plaques and tau pathology in the brain, producing learning and memory deficits, now widely used in the pathology of AD [32]. Female 3 × Tg-AD mice were selected for the study because of the faster progression of AD pathology in female mice compared with male mice [33]. The mode of administration, as well as the timing, is shown in the Figure (Fig. 4A), and at the end of the administration, the NOR test was performed. As shown in Fig. 4B, trajectory plots of the movements of mice in different subgroups were displayed (Fig. 4B). As we expected, compared with the WT group, mice in the 3 × Tg-AD group showed lower desire to explore and average speed on the first day, but the desire to explore improved significantly after administration of Ped treatment (Fig. 4C, D). After replacing one of the items on the second day, mice in the Ped administration group showed a significant preference for the new item. In contrast, the 3 × Tg-AD group showed a decreased ability to recognize the new object (Fig. 4E, F). These data further suggest that the cognitive of 3 × Tg-AD mice were significantly improved after Ped administration.

**Fig. 5:**
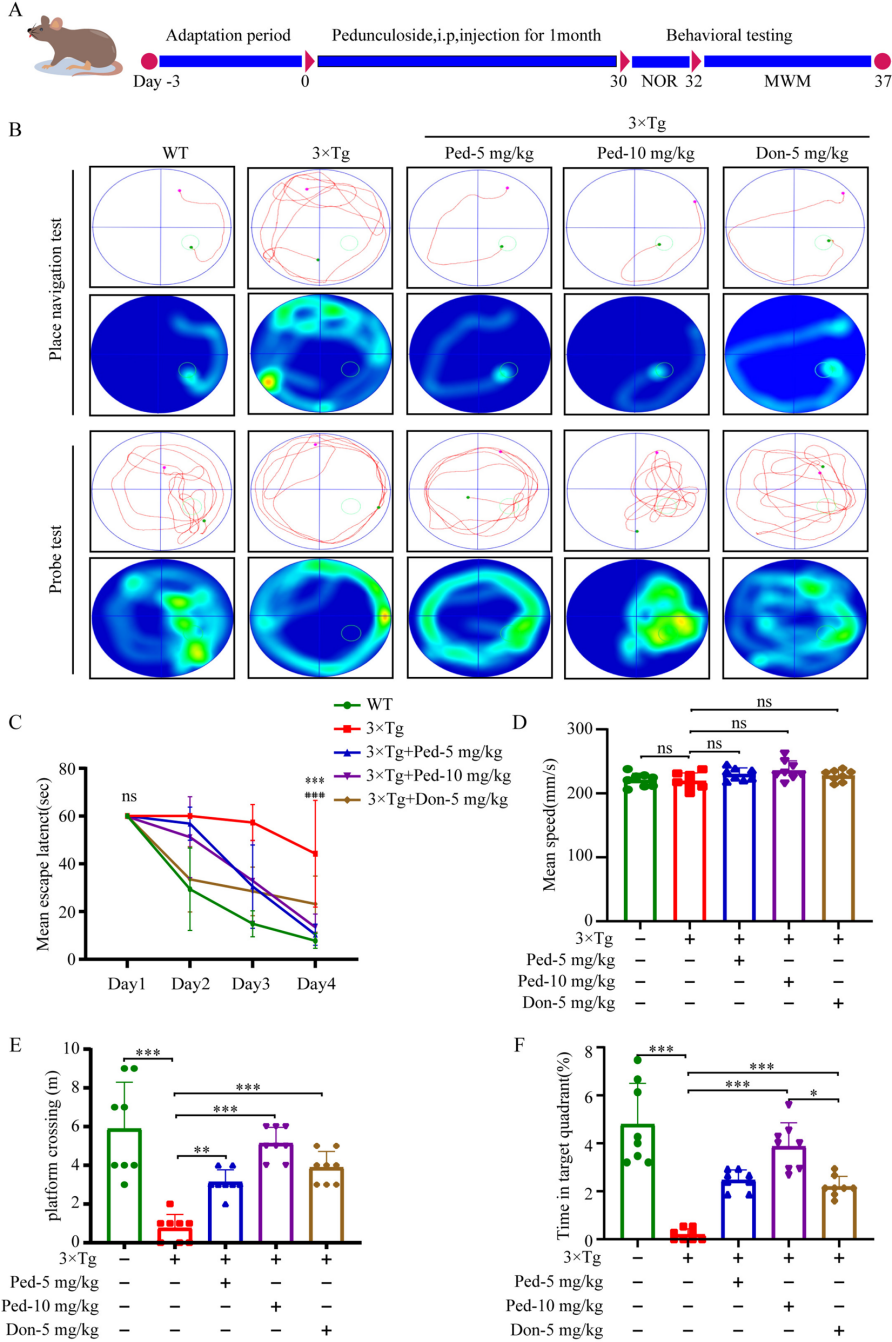
Ped ameliorates learning and memory deficits in 3 × Tg-AD mice. **A** Ped improved learning and memory in 3 × Tg-AD mice 8–month-old 3 × Tg-AD mice were administered Ped or donepezil by intraperitoneal injection once a day. **B** Track map of the activities of the place navigation test and the spatial probe test. **C** The escape latency results from the Morris water maze test on days 1–4. * represents the 3 × Tg-AD group compared to the WT group, # represents the drug group compared to the 3 × Tg-AD group. **D** Mean speed of movement of mice in each group during testing. **E** The average crossing platform times of each group of mice were within 60 s on day 5. **F** Time spent in the target quadrant where the platform had been located on day 5. *ns*—not significant, *P < 0.05 or **P < 0.01, or ***P < 0.001. n = 10 mice/Group

The day following the NOR experiment, we performed the MWM. The trajectories of mice in the MWM are shown in Fig. 5B. First, the mean escape latency of mice in the drug group was significantly shorter compared with that of mice in the model group (Fig. 5C). Similarly, there was no significant difference in the average swimming speed of the mice in each group (Fig. 5D); therefore, the results of the MWM reflect the therapeutic effect of Ped on memory impairment in 3 × Tg-AD mice. Compared with the model group, significant increases in the target quadrant time and the number of platform traversals were observed in mice in the administered group, and the therapeutic effect of Ped was significantly better than that of the positive drug donepezil (Fig. 5E, F). The 3 × Tg-AD mice exhibited significantly shorter target quadrant time and fewer plateau traversals compared to WT mice, which was improved by the administration of the Ped drug. Thus, the above results suggest that Ped can restore learning and memory deficits in mice in the 3 × Tg-AD experimental model.

### Ped treatment reduced AD pathology in 3 × Tg-AD mice brains

**Fig. 6:**
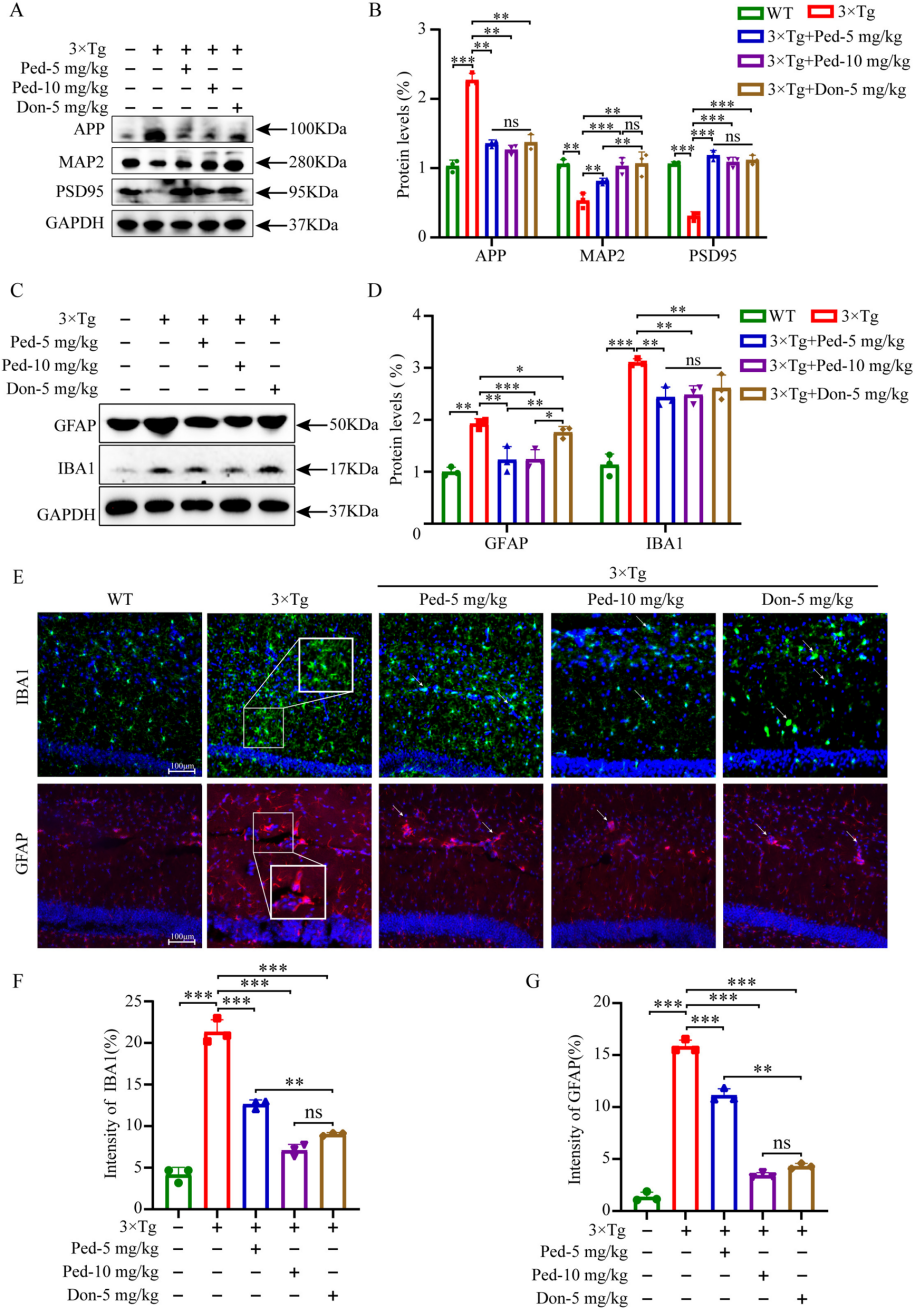
Ped alleviates AD pathology in 3 × Tg-AD mice. **A** Representative Western blot analysis of APP, MAP2, and PSD95 in the hippocampus. GAPDH was used as the loading control. **B** Quantitative analysis of the blot intensity of proteins in A were determined by Image J software. **C** Representative Western blotting analysis of IBA1, GFAP in the hippocampal homogenate. **D** Quantitative analysis of blot intensities of proteins in C were determined by Image J software. **E** Representative images of IBA1,GFAP immunofluorescence in the hippocampus CA1 of 5 groups mice at 8 months old. Scale bar 100 μm. **F**-**G** Quantitative analysis of (**E**). *ns*—not significant, *P < 0.05 or **P < 0.01, or ***P < 0.001. n = 3 independent experiments

The amyloid plaques consist of aggregates of Aβ produced by cleavage of amyloid precursor protein (APP) by β- and γ-secretase [34]. The accumulation of Aβ in the brain is a major trigger for subsequent pathological events, such as oxidative stress, neuroinflammation, and neuronal damage. Aβ stimulates microglia to transform into an activated state, releasing various inflammatory factors, which in turn leads to the activation of astrocytes [35, 36]. To determine whether Ped could attenuate the inflammatory response, we examined the expression of Ionized calcium-binding adaptor molecule 1 (IBA1) and Glial fibrillary acidic protein (GFAP) using immunofluorescence staining and Western blotting, respectively. The results showed that the activation of microglia and astrocytes in the hippocampus of model mice was significantly elevated in the model group compared with the WT group, whereas the activation of microglia and astrocytes was significantly reduced after treatment with Ped (Fig. 6C and E). Western blot for IBA1 and GFAP protein expression also showed the similar results (Fig. 6C).

To further evaluate the therapeutic effect of Ped on AD pathology, we examined the deposition of APP/β-Amyloid in the hippocampal region of mice using Western blot. As shown in Fig. 6A, treatment with Ped significantly reduced APP/ β-Amyloidprotein expression in the brains of 3 × Tg-AD mice.

During neuroinflammation, the co-activation of pro-inflammatory agents and associated cytotoxic products alters synaptic proteins, which in turn cause damage to neurons [37]. In AD, neuronal synaptic plasticity is a closely related indicator of learning and memory capacity. To investigate whether Ped could alleviate the impairment of synaptic plasticity, we examined the protein expression of Postsynaptic density protein 95 (PSD95) and Microtubule-associated protein 2 (MAP2) [38, 39]. As shown in Fig. 6A, prominence function was severely impaired in the model group of mice compared to the WT group. We found that treatment with Ped significantly alleviated the decrease in the levels of both proteins in 3 × Tg-AD mice. We get the conclusion that Ped can rescue impaired synaptic plasticity in 3 × Tg-AD mice.

### Ped reduces neuronal apoptosis in the *hippocampus* of 3 × Tg-AD mice by activating the AMPK signaling cascade

**Fig. 7:**
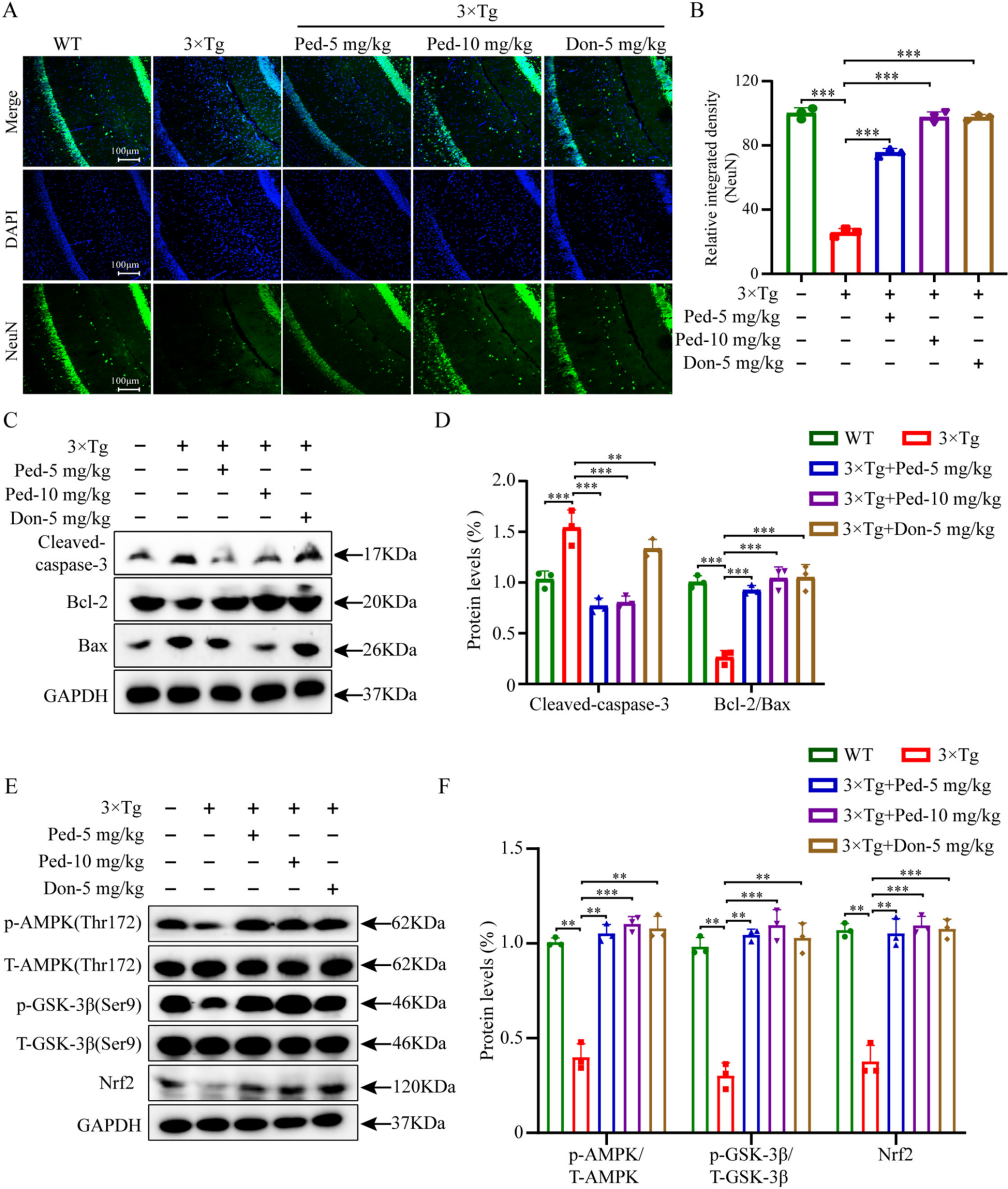
Ped treatment reduces neuronal apoptosis in 3 × Tg-AD mice. **A** Representative images of NeuN staining. **B** Quantitative analysis of (**A**). **C** Representative Western blot analysis of Cleaved-caspase-3, Bax, and Bcl-2 in the hippocampus. GAPDH was used as the loading control. **D** Quantitative data of the blot intensity of corresponding proteins were determined by Image J software. **E** Representative Western blot analysis of p-AMPK, T-AMPK; p-GSK-3β, T-GSK-3β, and Nrf2 in the hippocampus. **F** Quantitative data of the blot intensity of corresponding proteins were determined by Image J software. *ns*—not significant, *P < 0.05 or **P < 0.01, or ***P < 0.001. n = 3 independent experiments

To investigate the influence of Ped on apoptosis in the hippocampus of 3 × Tg-AD mice, we utilized Western blot analysis to assess the protein expression of Bax, Bcl-2, and Cleaved-caspase-3 (Fig. 7C). Remarkably, the high dose of Ped administered to the 3 × Tg-AD model mice resulted in a significant upregulation of Bcl-2/Bax expression. Notably, Ped effectively suppressed the elevation of Cleaved-caspase-3 protein expression in the hippocampus of 3 × Tg-AD model mice, indicating its favorable anti-apoptotic capability. To further validate the above results, we examined the neuronal damage in the CA1 region of the hippocampus of the mouse brain using NeuN immunofluorescence (Fig. 7A). Interestingly, we observed a marked reduction in neurons in the 3 × Tg-AD group compared to the WT group; however, administration of a high dose of Ped reversed the neuronal decline in various brain regions (Fig. 7B).

Overall, based on the results of the in vitro study, we found that Ped exerts its protective effect on PC12 cells by activating the AMPK signaling cascade. Based on the results obtained in vitro, we investigated in vivo whether Ped treatment was also activated through this pathway. Figure 7E supports our hypothesis that the AMPK/GSK-3β/Nrf2 signaling pathway is activated in the hippocampus after Ped-based treatment (Fig. 7F). Therefore, we can conclude that Ped exerts its anti-apoptotic and antioxidant effects by activating the AMPK/GSK-3β/Nrf2 pathway.

## Discussion

In the present study, we found that Ped could reduce Aβ-induced apoptosis in PC12 cells by increasing mitochondrial membrane potential and reducing ROS. In terms of mechanism, we demonstrated that Ped exerts anti-oxidative stress and anti-apoptosis effects in part by activating the AMPK signaling cascade. This conclusion was further verified by using the AMPK inhibitor compound C. In vivo, we found that the spatial memory ability of 3 × Tg-AD mice was significantly improved after Ped treatment. Furthermore, Ped reduced astrocyte and microglia activation and Aβ aggregation. Similarly, Ped reduces neuronal apoptosis in 3xTg-AD mice through the AMPK/GSK-3β/Nrf2 signaling pathway. These data suggest that Ped may be a potential drug for the treatment of AD.

Due to the complex and multifactorial pathology of AD, Traditional Chinese Medicine can be used as an alternative treatment method, which contains multiple active ingredients that can act on multiple targets at the same time to produce a cumulative or synergistic effect [40]. The use of Traditional Chinese Medicine for treating AD has been in clinical practice for many years [41]. Research on the mechanism of drug action will help to reveal the therapeutic targets of Traditional Chinese Medicine intervention in AD, thus providing a scientific basis for drug therapy. Saffron can enhance blood oxygen supply and effectively improve memory [42]. Bacopa monnieri improves memory by enhancing cholinergic function by inhibiting acetylcholinesterase [43]. Poria cocos, Ganoderma lucidum, and Sophora flavescens have immune-modulating functions. These drugs are used to treat stroke, cerebral ischemia, and diabetes [44–46]. In addition, many bioflavonoids have strong antioxidant effects. Ped is a traditional Chinese herb extracted from *Cortex Ilicis Rotundae* [15, 19]. Modern pharmacology has shown that Ped has a variety of pharmacological activities, such as prevention and treatment of rheumatoid arthritis [17], improvement of dyslipidemia in high-fat diet-induced hyperlipidemic rats [47], improving intestinal flora [48], and other functions. In the present study, we demonstrate for the first time that Ped can exert anti-inflammatory and anti-apoptotic effects to slow down AD pathology through the AMPK/GSK-3β/Nrf2 signaling pathway.

Cerebral neurons are vulnerable to oxidative damage due to their high content of oxidability polyunsaturated fatty acids and their high oxygen consumption. However, the brain's antioxidant system exhibits comparatively limited efficacy in countering this damage [49]. Unlike other cell types, neurons have limited nutrient storage capacity but exhibit highly active metabolism, making them particularly susceptible to energy fluctuations [50]. AMPK plays a crucial role in numerous diseases. Activation of AMPK affects systemic energy intake and energy expenditure and thus may be an important target for the treatment of obesity [51]. In obese mice, AMPK activity is reduced, and because AMPK increases muscle uptake of glucose, AMPK has been recognized as a potential target for diabetes treatment [52]. It plays an important role in diseases such as non-alcoholic steatohepatitis, inflammatory bowel disease, myotonic dystrophy, neuronal loss, and many others [53]. Similarly, AMPK dysfunction is associated with neurodegenerative diseases including AD. To protect against the damage caused by oxidative stress, neurons activate endogenous antioxidant defense systems such as Nrf2 to reduce oxidative damage [54]. Among all antioxidant proteins, Nrf2 is very important as it protects the brain from various oxidative stress-induced damages [55]. Moreover, through the activation of Nrf2, neuronal cultures have shown protection against amyloid-β-induced neurotoxicity [56]. Numerous studies have provided evidence supporting the notion that in patients with AD, there is a decrease in Nrf2 activation and/or impaired nuclear translocation. Glycogen synthase kinase-3 (GSK-3) is a serine-threonine kinase that primarily phosphorylates and deactivates glycogen synthase, thereby modulating glycogen synthesis [57]. GSK-3 has both α and β isozymes and both share a high degree of amino acid homology [58]. GSK-3β assumes a pivotal function in facilitating mitochondrial function and experiences inhibition through phosphorylation at Ser9. Previous findings have shown that AMPK inhibits GSK-3β activity and further promotes Nrf2 accumulation in the nucleus, leading to antioxidant response element (ARE)-driven gene transcriptional activation, which in turn restores cellular antioxidant capacity [59]. In the present study, we found that treatment with Ped increased phosphorylation of GSK-3β at the Ser9 site both in Aβ-induced PC12 cells and in 3 × Tg-AD model mice. Our results suggest that treatment with Ped can increase the phosphorylation of AMPK, GSK-3β Ser9 site, and enhance the expression level of Nrf2 protein, thus exerting neuroprotective effects.

A temporal correlation has been observed between the neuroprotective effects induced by Ped in insulted neurons and the neurotherapeutic benefits observed in the 3 × Tg-AD mouse model. This correlation is accompanied by the stimulation of AMPK/GSK-3β phosphorylation activity and upregulation of activated Nrf2 expression. These findings suggest that Ped may have the potential to serve as a novel activator of AMPK/GSK-3β/Nrf2 pathway, providing therapeutic effects against inflammation and oxidative stress. In addition, it can be used as a potential drug for AD treatment. Pre-treatment with Ped effectively mitigated Aβ-triggered apoptosis, reduced ROS levels, and restored mitochondrial membrane potential. The outcomes of both in vivo and in vitro investigations demonstrated the antioxidant and neuroprotective effects of Ped. However, there are some flaws in our experiments, such as we have not yet clarified the direct target of action of Ped. We will continue to explore the direct targets of Ped in the future and improve its molecular mechanism.

## Conclusion

In conclusion, Ped attenuated Aβ pathology, reduced neuronal apoptosis and neuroinflammation, and ultimately improved cognitive dysfunction in 3 × Tg-AD mice. Mechanistically, we found that Ped exerted neuroprotective effects by activation of the AMPK signaling cascade. Importantly, the efficacy of Ped was superior to that of the positive control drug donepezil. Our results support that Ped is a potential drug for AD therapy.

## Supplementary Information

Additional file 1.

## Data Availability

The data used to support the findings of this study are included within the article.

## List of abbreviations

Aβ: Amyloid-beta
AD: Alzheimer's disease
AMPK: AMP-activated protein kinase
APP: Amyloid precursor protein
DMEM: Dulbecco’s modified Eagle’s medium
DAPI: 4’*,6-diamidino-2’-phenylindole*
FBS: Fetal bovine serum
GSK-3β: Glycogen synthase kinase-3β
GFAP: Glial fibrillary acidic protein
IF: Immunofluorescence
IBA1: Ionized calcium-binding adaptor molecule 1
MAP2: Microtubule-associated protein 2
MWM: Morris water maze
NeuN: Neuron-specific nuclear protein
Nrf2: Nuclear factor erythroid-2-related factor 2
NOR: New Object Recognition
NFTs: Neurofibrillary tangles
PSD95: Postsynaptic density protein 95
PBS: Phosphate-buffered saline
ROS: Reactive oxygen species
