# Supplementary material for: Pedunculoside alleviates cognitive deficits and neuronal cell apoptosis by activating the AMPK signaling cascade
Source: Chin Med. 2024 Nov 22;19:163. doi: 10.1186/s13020-024-01033-6 (PMC11583384; doi:10.1186/s13020-024-01033-6)
Supplement: Supplementary file 1 — Additional file 1. [file 13020_2024_1033_MOESM1_ESM.docx]

**Supplemental Information**

**Table S1. Antibodies used in this study**

| **Antibody** | **Cat. NO** | **Source** | **Dilution** |
| --- | --- | --- | --- |
| MAP2 | 4542 | CST | WB:1:1000 |
| PSD95 | 2507 | CST | WB:1:1000 |
| Iba1 | 17198 | CST | WB:1:1000/IF:1:100 |
| GFAP  GFAP(GA5) | 3670 | CST | WB:1:1000/IF:1:200 |
| APP | 2450 | CST | WB:1:1000IF:1:200 |
| Phospho-AMPKα (Thr172)  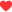  关注商品（0人气）  **Phospho-AMPKα (Thr172)**  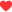  关注商品（0人气）  **Phospho-AMPKα (Thr172)** | 2535 | CST | WB:1:1000 |
| AMPK | 2532 | CST | WB:1:1000 |
| Phospho-GSK-3β (Ser9) | 5558S | CST | WB:1:1000 |
| GSK-3β (Ser9) | db14808 | Diagbio | WB:1:2000 |
| Nrf2 | 12721 | CST | WB:1:1000 |
| Bax | 2774 | CST | WB:1:1000 |
| Bcl2 | 15071 | CST | WB:1:1000 |
| NeuN | 24307 | CST | IF:1:200 |
| Cleaved-caspase-3 | 9661 | CST | WB:1:1000 |
| GAPDH | AF0006 | Beyotime | WB:1:2000 |
| Anti-rabbit IgG HRP | A0208 | Beyotime | WB:1:2000 |
| Anti-Mouse IgG HRP | A0216 | Beyotime | WB:1:2000 |
| Alexa Fluor® 594 | 8889 | CST | IF: 1:500 |
| Alexa Fluor® 488 | 4412 | CST | IF: 1:500 |

**Table S2. Natural compound used in this study**
